# Quantum Oscillations from Nontrivial States in Quasi-Two-Dimensional Dirac Semimetal ZrTe_5_ Nanowires

**DOI:** 10.1038/s41598-019-39144-y

**Published:** 2019-03-05

**Authors:** Pei Yang, Wei Wang, Xiaoqian Zhang, Kejie Wang, Liang He, Wenqing Liu, Yongbing Xu

**Affiliations:** 1 Jiangsu Provincial Key Laboratory for Nanotechnology, Collaborative Innovation Center of Advanced Microstructures, School of Electronic Science and Engineering, Nanjing University, Nanjing, 210093 P. R. China; 2 York Nanjing Joint Centre for Spintronics and Nanotechnology, Departments of Electronics, The University of York, York, YO10 5DD UK

## Abstract

Recently discovered Dirac semimetal ZrTe_5_ bulk crystal, exhibits nontrivial conducting states in each individual layer, holding great potential for novel spintronic applications. Here, to reveal the transport properties of ZrTe_5_, we fabricated ZrTe_5_ nanowires (NWs) devices, with much larger surface-to-volume ratio than bulk materials. Quantum oscillations induced by the two-dimensional (2D) nontrivial conducting states have been observed from these NWs and a finite Berry phase of ~π is obtained by the analysis of Landau-level fan diagram. More importantly, the absence of the Aharonov-Bohm (A-B) oscillations, along with the SdH oscillations, suggests that the electrons only conduct inside each layer. And the intralayer conducting is suppressed because of the weak connection between adjacent layers. Our results demonstrate that ZrTe_5_ NWs can serve as a suitable quasi-2D Dirac semimetal with high mobility (~85000 cm^2^V^−1^s^−1^) and large nontrivial conductance contribution (up to 8.68%).

## Introduction

The simple binary compound ZrTe_5_ is known as a thermoelectric material with layered structure. Recently it has been theoretically predicted as a 2D TI with a bulk bandgap of 0.4 eV^1^ in the single layer form, while its three-dimensional (3D) version has been reported with many contradictory results such as 3D TI^2,3^, 3D strong TI^4^ and quasi-2D or 3D Dirac semimetal^5–9^. Our previous study has suggested that the transport property of bulk ZrTe_5_ is a collective behavior of many individual monolayers^10^. However, the critical transport phenomena in NWs which have a much larger surface-to-volume ratio comparing to their bulk forms have not been fully studied.

Here, we have fabricated ZrTe_5_ NWs devices to probe the electric transport from the nontrivial conducting states. We report the Shubnikuv-de Hass (SdH) oscillations originated from the nontrivial states of each monolayer. The SdH interactions reveal a well-defined 2D Fermi surface lasting up to 20 K. The finite Berry phase of ~π with a high mobility of 85000 cm^2^V^−1^s^−1^ clarifies the topological nontrivial nature. More importantly, the A-B oscillations were not observed when the magnetic field was applied along the current direction parallel to the NW. All of these suggest that the electrons only conduct within each layer and cannot hop between different layers, since the interlayer coupling between adjacent layers is too weak. According to the calculation by H. Weng *et al*., the binding energy of ZrTe_5_ is only 12.5 meV, much lower than Bi_2_Se_3_ (27.6 meV) and very close to graphite (9.3 meV)^1^.

## Results

### Morphology and structural characterization of ZrTe_5_ NWs

**Figure 1:**
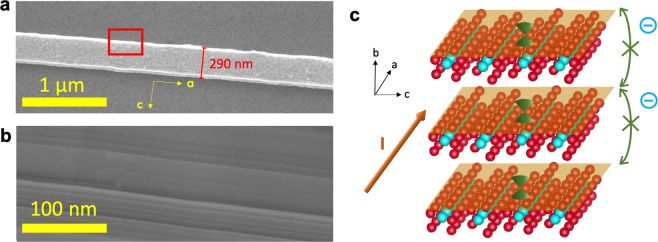
Morphology characterization of ZrTe_5_ NWs. (**a**) SEM image of a ZrTe_5_ NW, with a diameter of ~290 nm. (**b**) A 30° tilt SEM zoom-in image of the rectangular frame in a. It clearly shows steps on the side (ab plane). (**c**) A schematic diagram of nontrivial conducting states existing on each layer of ZrTe_5_. The yellow sheets represent the conducting states.

Figure 1a exhibits the scanning electron microscope (SEM) image of a ZrTe_5_ NW with a diameter of ~290 nm. The length of our ZrTe_5_ NWs ranges from a few microns to tens of microns. Figure 1b is the tilted SEM of the NW with a tilt angle of 30°. It shows clearly the layer steps on the side walls.

**Figure 2:**
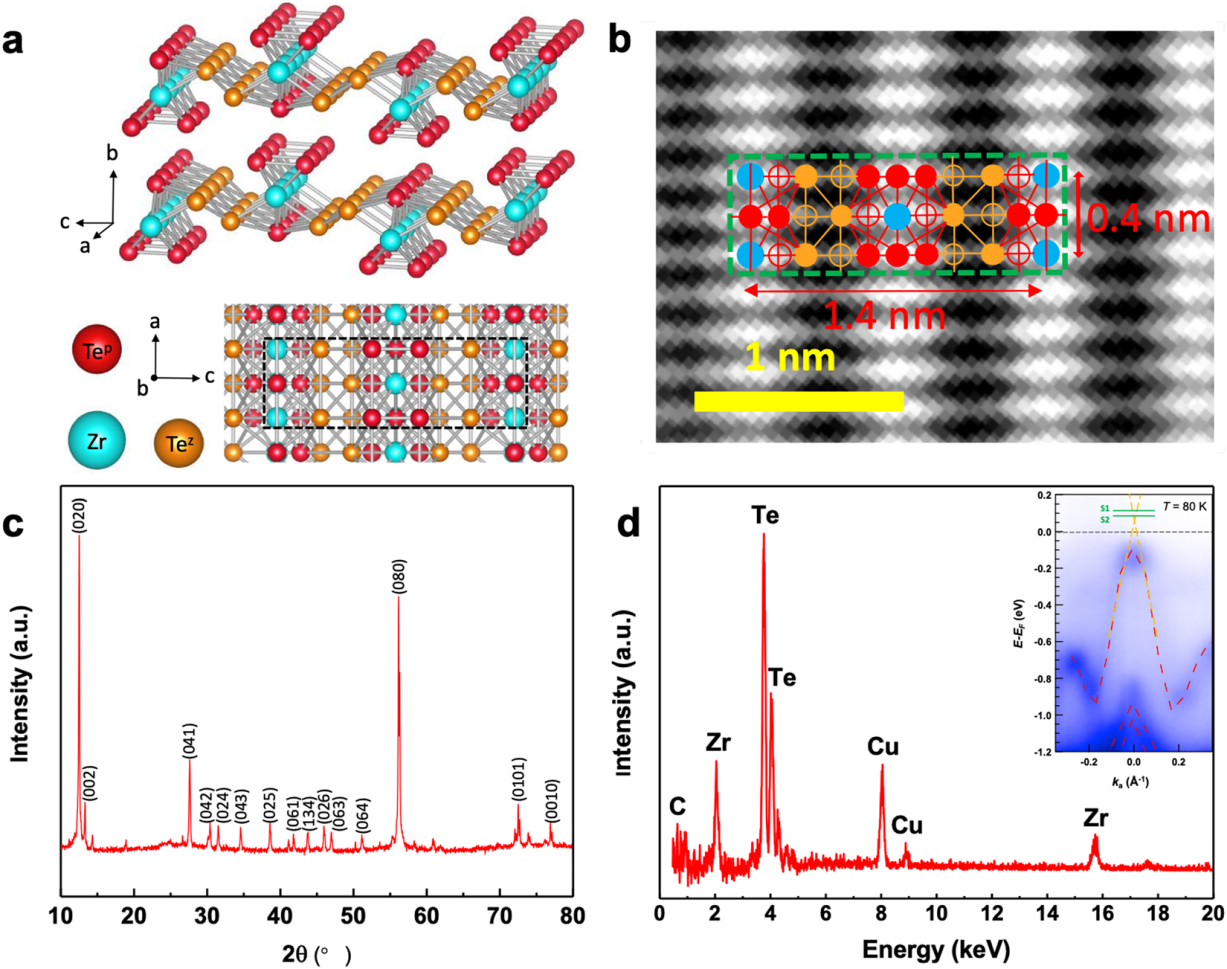
Structural characterization of ZrTe_5_ crystal. (**a**) Top: Tilt view of the crystal structure of ZrTe_5_ layers. The blue spheres represent Zr atoms and the red and orange spheres represent the prismatic (Te^p^) and the zigzag Te (Te^z^) atoms, respectively. Bottom: Top view of the crystal structure of two ZrTe_5_ layers. Atoms at the top layer are brighter, and that at the bottom layer are dimmer. The black dashed rectangular marks one unit cell. (**b**) TEM image of a ZrTe_5_ NW on a holey carbon grid reveals a perfect crystalline structure. The unit cell is shown in dashed green rectangular. The solid and open symbols represent atoms in the top and the second layers. Blue, red and orange circles represent Zr, Te^p^ and Te^z^, respectively. The bright spots are due to the overlapping of Zr and Te atoms in the projection. The measured lattice constants are a = 0.4 nm and c = 1.4 nm. (**c**) X-ray powder diffraction pattern of ZrTe_5_ NWs. All the peaks can be indexed by ZrTe_5_ crystal, and the calculated lattice constants are a = 0.398 nm, b = 1.452 nm, and c = 1.372 nm. (**d**) EDX spectrum of ZrTe_5_ NWs, Zr and Te are the only elements that can be detected, except C and Cu. The inset shows the ARPES image of bulk ZrTe_5_ crystal at 80 K. For comparison, the calculated band structure is plotted on top of the experimental data (red dashed curves). The green lines represent the *E*_F_ of S1 and S2 obtained below, respectively.

The crystallographic structure of ZrTe_5_ is an orthorhombic layered structure^11^ which is shown in Fig. 2a. Prism chains of ZrTe_3_ (Te^p^) is along the a-axis, and these prismatic chains are bonded via zigzag Te atoms (Te^z^) along the c-axis to form a 2D sheet of ZrTe_5_ in the a-c plane. The sheets of ZrTe_5_ form a layered structure stacking along the b-axis. The primitive unit cell contains two formula units with two prismatic chains and two zigzag chains, as indicated by the dashed black square in Fig. 2a.

To perform the transmission electron microscopy (TEM)-energy dispersive X-ray spectrometry (EDX) analyses, the ZrTe_5_ crystal NWs were dispersed in ethanol and deposited onto a carbon film supported by a 200 mesh, 3 mm diameter copper grid. Figure 2b reveals the atomic level details of the a-c plane of ZrTe_5_. From the TEM image, we are able to determine the lattice constants of a and c are 0.4 nm and 1.4 nm, respectively. At the same time, from the X-ray diffraction (XRD) pattern (Fig. 2c), we can calculate the lattice constants a = 0.398 nm b = 1.452 nm and c = 1.372 nm, consistent with the result of TEM. The EDX spectrum detected Zr, Te, C and Cu, which were attributed to the NWs, the carbon film and copper grid (Fig. 2d). The Zr/Te atomic ratio in the EDX area was 0.28, which was much lower than that of the nominal composition. This discrepancy is probably due to the vacancies of Te atoms, similar to those reported in 3D TI, Bi_2_Te_3_ ^12^. The band dispersion obtained by ARPES measurement of bulk ZrTe_5_ crystal^8^ at 80 K is sketched in the inset of Fig. 2d. An almost linear E-K dispersion (as indicated by the yellow dashed lines) was observed near the Γ point, suggesting the presence of Dirac fermions. Moreover, the energy dispersion shows a small gap opening rather than massless Dirac cone. Note that our ARPES result was done at 80 K, and the band diagram is going to shift down as the temperature decreases to lower values of 2–20 K^13^, which results in the *E*_F_ of our samples (S1 and S2) shifting up as indicated by the green lines at lower temperatures similar to that from the ref.^2^, as estimated from the quantum oscillations. The temperature dependent shift of *E*_*F*_ is probably due to the change of lattice constants^1,14^, as the temperature decreases.

### Electrical transport measurements of ZrTe_5_ NW devices

**Figure 3:**
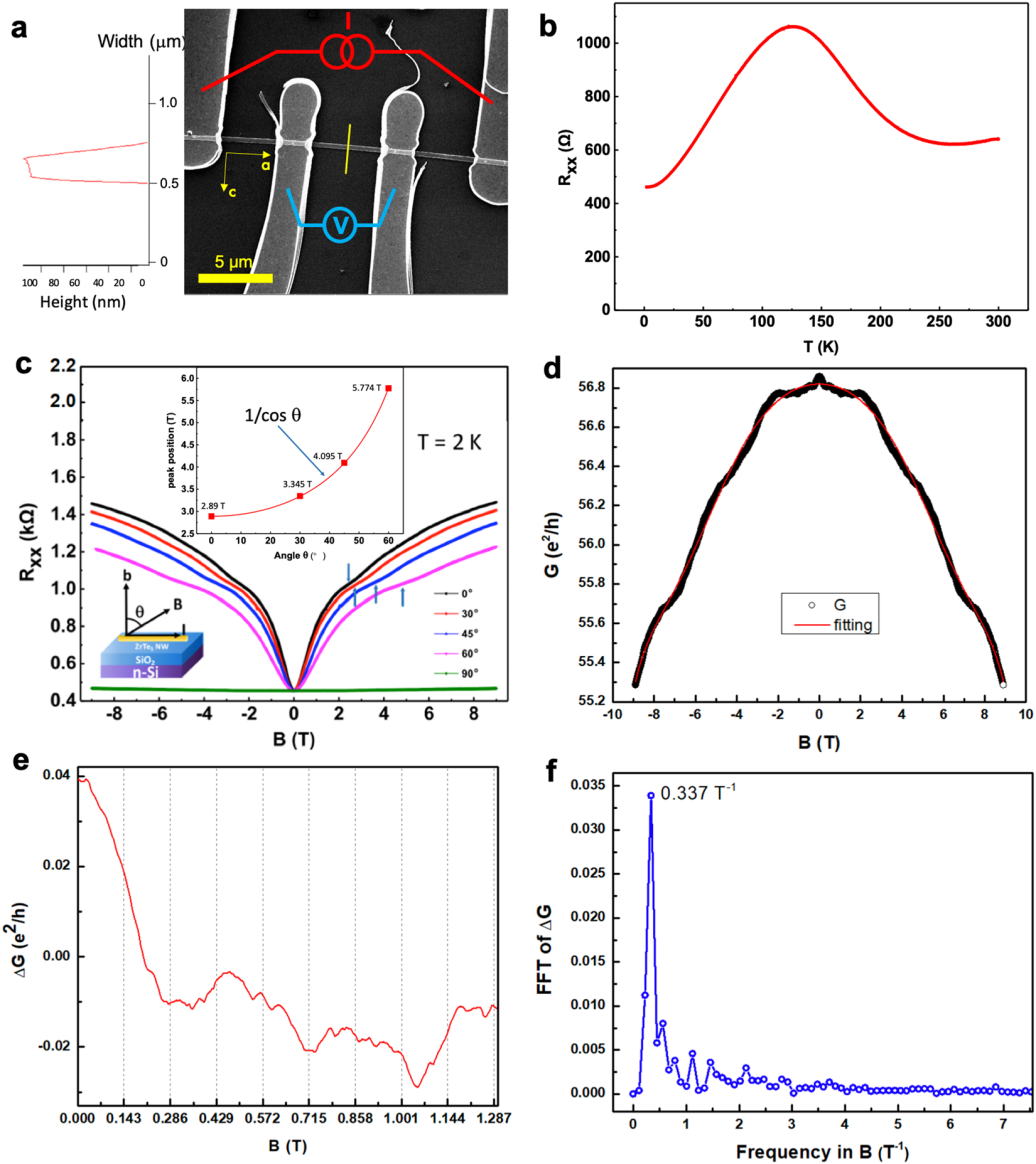
Electrical transport measurements of ZrTe_5_ NW devices. (**a**) An SEM image of the ZrTe_5_ NW device with four Au contacts. The channel length is 4.6 μm. The thickness of the NW is ~100 nm, measured by AFM (**a** line cut in the inset, indicated with the yellow short line in the SEM image). The crystallographic a- and c-axis are shown by two arrows, respectively. (**b**) Temperature dependent longitudinal resistance. A resistance peak at ~125 K can be observed. (**c**) The field dependent resistance at different tilt angle *θ*. Oscillations can be seen from the raw data. The inset shows the field position of the n = 3 LL valley for sample S1 (blue arrows in **c**) varies with *θ* as 1/cos*θ* (red curve), consistent with a 2D FS. (**d**) Zoom-in image of the magneto-conductance in the unit of *e*^2^/*h* at *θ* = 90° (black dots) and the background of a polynomial fit (red solid line). (**e**) The magneto-conductance after subtracted the smooth polynomial, shows no A-B oscillations with a period of 0.143 T. (**f**) FFT of the Δ*G* curve with a peak at 0.337 T^−1^ marked on the image.

Figure 3a exhibits a four-probe device with Au contacts while the width and channel length are 3.2 µm and 4.6 µm, respectively. The temperature dependent longitudinal resistance *R*_xx_ of S2 is shown in Fig. 3b. *R*_xx_ demonstrates a peak at 125 K, known as the “resistivity anomaly”, close to the values reported in previous work (~60–170 K). This resistivity anomaly might be associated with a change in the electronic structure caused by thermal expansion, and the detailed physics is still under debating^2–4,15–30^.

As we know, one of the persuasive proofs for characterizing nontrivial conducting states is regarded as SdH oscillations^8,29–32^. According to this, low-temperature magneto transport measurements were carried out to demonstrate the nontrivial conducting states in ZrTe_5_ NWs experimentally.

The angle dependent magneto transport properties of S1 are shown in Fig. 3c,d. Figure 3c exhibits the field dependent resistance for different *θ* (0°, 30°, 45°, 60° and 90°) from −9 T to + 9 T at 2 K before subtracting the smooth background. Here *θ* is the tilt angle between the field direction ***B*** and the crystallographic b-axis, within b-a plane. The magnetoresistance, MR curves display pronounced SdH oscillations from 0° to 60°. While MR(*B*) = (*R*(*B*) − *R*(0))/*R*(0), typically has a magnitude of 321% near 9 T, the amplitude of the SdH signal is small and amounts to only 0.4% of the total resistance. After subtracting a smooth background, Δ*R*_xx_ demonstrates much more clear oscillations. The inset of Fig. 3c shows the magnetic field *B* corresponding to the *n* = 3 minimum at varies rotation angles *θ*, up to 60°. It follows 1/cos(*θ*) perfectly. This concludes that the quantum oscillations arise from a 2D Fermi surface.

### Quantum oscillations arising from the 2D nontrivial states

**Figure 4:**
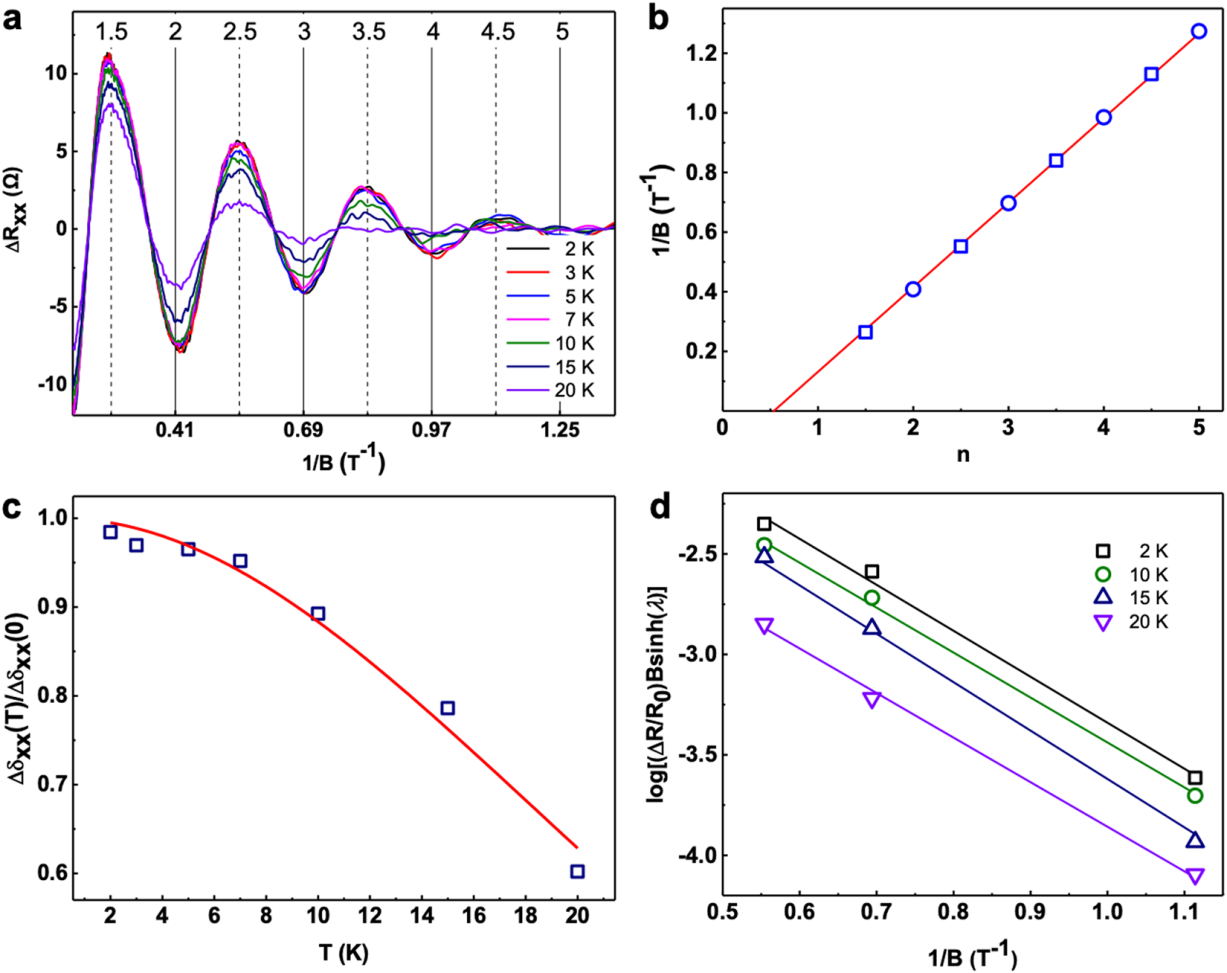
Quantum oscillations arising from the 2D nontrivial states. (**a**) Temperature dependent SdH oscillations of ZrTe_5_ NWs at *θ* = 0°. The black solid lines mark the SdH valleys at Landau filling factors of 2, 3, 4 and 5, while the dash lines mark the peaks at 1.5, 2.5, 3.5 and 4.5. (**b**) Landau-level fan diagram. Linear fitting gives a nonzero intercept of 0.580, corresponding to a Berry phase of ~*π*. (**c**) Temperature dependence of the normalized conductivity amplitude Δ*σ*_xx_(*T*)/Δ*σ*_xx_(0). The solid red line is the best fit to *λ*(*T*)/sinh(*λ*(*T*)). A magnetic field of 5.22 T was used to extract the cyclotron effective mass: ~0.031 *m*_e_. (**d**) Dingle plots of log [(Δ*R*/*R*_0_)*B*sinh(*λ*)] versus 1/*B* at four different temperatures. Transport lifetime *τ*, mean free path *l* = *V*_F_*τ*, and mobility *μ* can be extracted from the best fit to log [(Δ*R*/*R*_0_)*B*sinh(*λ*)].

The temperature dependent magneto transport properties of S2 after subtracting a smooth background is shown in Fig. 4a. The oscillatory part of *R*_xx_ (Δ*R*_xx_) reveals periodic dependences with peaks (maxima) and valleys (minima) versus 1/*B*, indicating there’s a well-defined Fermi surface^31,33,34^. The magnetic field is perpendicular to both the c-axis and the charge current flow (a-axis) of the ZrTe_5_ NW (*θ* = 0°). The SdH oscillations can be seen from 2 K up to 20 K. After fast Fourier transform (FFT) we can obtain a single oscillation frequency (*f*_SdH_(*T*), 3.57 T). For a 2D system, the Onsager formula: *f*_SdH_ = (*h*/4π^2^*e*)*S*_F_, can describe the relation between SdH oscillation frequency and the cross section of the Fermi surface (*S*_F_), where *S*_F_ = π*k*_F_^2^, *k*_F_ is the Fermi vector, *e* is the electron charge, and *h* is Planck constant. The 2D surface carrier density (*n*_2D_) can be calculated by *n*_2D_ = *k*_F_^2^/4π. Then we can extract *k*_F_ to be 0.0104 Å^−1^ by substituting *S*_F_ in *f*_SdH_, corresponding to *n*_2D_ = 0.86 × 10^11^ cm^−2^.

The 1/*B* values of the maxima (hollow rectangles) and the minima (hollow circles) in Δ*R*_xx_ versus Landau level index *n*^35^ are plotted in Fig. 4b. We extracted a finite intercept of 0.580 ± 0.001, by linear fitting of the data, indicating a Berry phase of ~π, emphasizing the topological nature of the SdH oscillations. We have noticed that there’s a little discrepancy between the intercept and 1/2. The reason could be that for 3D or quasi-2D crystal, there is an additional phase shift determined by the dimensionality of the Fermi surface and the value changes from 0 for surface states (2D) to ± 1/8 for bulk states (3D)^36^. Such inconsistence may also be attributed to the Zeeman splitting and/or the multiple Hall-channel contributions^37^.

The temperature-dependent amplitude of Δ*σ*_xx_ can be written as Δ*σ*_xx_(*T*)/Δ*σ*_xx_(0) = *λ*(*T*)/sinh (*λ*(*T*)), where *λ*(*T*) = 2π^2^*k*_B_*Tm*_cycl_/(*ħeB*), *m*_cycl_ is the cyclotron mass, *ħ* is the reduced Planck’s constant, and *k*_B_ is Boltzmann’s constant. After fitting the conductivity oscillation amplitude to the Δ*σ*_xx_(*T*)/Δ*σ*_xx_(0) equation, *m*_cycl_ is calculated to be ~0.031 *m*_e_ (*m*_e_ is the free electron mass), which is shown in Fig. 4c. For a Dirac-like dispersion, *E*_F_ = *ħk*_F_*V*_F_ = *p*_F_*V*_F_ and also *p*_F_ = *m*_cycl_*V*_F_, so *m*_cycl_ = *E*_F_/*V*_F_^2,29,32,38^. This yields a Fermi level of ~26.64 meV above the Dirac point and a Fermi velocity of ~3.89 × 10^5^ m/s, which is in a good agreement with results reported by others^5,39^.

We can extract the transport lifetime of the surface states (*τ*) by the Dingle plot^30,31,33,40^. Since Δ*R*/*R*_0_ ~ [*λ*(*T*)/sinh*λ*(*T*)]*e*^−*D*^, where *D* = 2π^2^*E*_F_/*τeBV*_F_^2^, the lifetime *τ* can be obtained by the slope in Dingle plot by log[(Δ*R*/*R*_0_)*B*sinh(*λ*(*T*))] ≈ [2π^2^*E*_F_/(*τeV*_F_^2^)] × (1/*B*). The fit in Fig. 4d extracts a lifetime *τ* ~ 1.5 × 10^−12^ s, indicating a mean free path *l* of ~583 nm (*l* = *V*_F_*τ*). The surface mobility *μ*_s_ = *eτ*/*m*_cycl_ = *el*/*hk*_F_ can be estimated as ~85000 cm^2^ V^−1^ s^−1^ (see Table 1). Note that the high mobility in our ZrTe_5_ NW with a Zr/Te atomic ratio of 0.28 is reasonable since the 2D conducting states’ mobilities of topological materials are always very high. And for our samples, although the bulk have a lot of Te vacancies, the 2D conducting states are robust again those non-magnetic defects^3^. According to these results, the 2D nontrivial conducting states contribution to the total conduction can be calculated as ~8.68% (Table 2).

**Table 1:** Estimated parameters from the SdH oscillations at T = 2 K.

Sample	f_SdH_ (T)	n_2D_ (10^11^ cm^−2^)	m_cycl_ (m_e_)	k_F_ (Å^−1^)	V_F_ (10^5^ ms^−1^)	E_F_(meV)	τ(10^−12^ s)	l(nm)	µ(cm^2^ V^−1^s^−1^)
S1	8.58	2.08	0.037	0.0161	5.04	53.59	1.75	881	82915
S2	3.57	0.86	0.031	0.0104	3.89	26.64	1.5	583	85083

**Table 2:** Estimated surface conduction percentage with zero magnetic field and at T = 2 K.

Sample	G(SdH) (mS)	R(total) (Ω)	R_sheet_(total) (Ωϒ^−1^)	G_sheet_ (total) (mS)	G(SdH)/G_sheet_ (total)
S1	2.76	453.7	9.07	11.02	~2.50%
S2	1.18	460.9	73.74	13.56	~8.68%

### The absence of A-B oscillations

Quantum interference effects, such as A-B oscillations^41^ associated with the surface states may occur for mesoscopic samples where the low-temperature mean free path is comparable to the sample dimensions. Theoretically, only half revolution around the perimeter of the NW (~390 nm, since the thickness (*t*) and width (*w*) of our NW are 100 nm and 290 nm respectively) is required for the interference effect of the A-B oscillations. Practically the mean free path *l* extracted from our results is ~583 nm, which is a lower estimation of the phase-coherent diffusion length in general. So, the phase-coherent diffusion length is long enough for the observation of A-B oscillations in the NW, if it exists.

Further calculation indicates that the cross-sectional area of the NW is *S* = *w* × *t* = 2.9 × 10^−14^ m^2^. Thus, the characteristic period of the A-B oscillations should be Δ*B* = *Φ*_0_/*S* = 0.143 T, where *Φ*_0_ = *h*/*e* is the flux quantum, *S* is the cross-sectional area of the NW, *h* is Planck’s constant and *e* is the electron charge^32^. Meanwhile, we can estimate the amplitude of the A-B oscillations, if exist. The unsuppressed amplitude of A-B oscillations should be in the order of *e*^2^/*h* in conductance, which means when *G*_0_ ( = 1/*R*_0_) changes in *e*^2^/*h*, the resistance after changing should be 1/(1/*R*_0_ + *e*^2^/*h*), giving the Δ*R* = *R*_0_ − *R*. That is, Δ*R* = Δ(1/*G*) ≈ *R*_0_ − 1/(1/*R*_0_ + *e*^2^/*h*) ≈ 7.85 Ω, considering *R*_0_ ~ 454.1 Ω. However, for the magneto-conductance curves in the unit of *e*^2^/*h* at *θ* = 90° (*B*//*I*), there’s no A-B oscillations in our NWs (Fig. 3d).

After subtracted the smooth background (red lines in Fig. 3d), the magneto-conductance trace Δ*G* measured in a longitudinal field is shown in Fig. 3e. From this quantum magneto-conductance curve, there’s no such oscillations consistent with the calculated A-B oscillations whose period should be 0.143 T. Meanwhile, the upper bound of Δ*G* is ~0.04 *e*^2^/*h*.

However, A-B oscillations are usually suppressed in cylindrical conductors, like our NWs. The main origins of this reduction are probably as follows. First, different slices of the metal cylinder (effectively 2D metal ring) generate A-B oscillations of random phases, canceling each other. Second, the electrons circle along the NW perimeter in a quasi-ballistic manner, but drift along the longitudinal direction of the NW in a diffusive manner (mean free path < the length of NW, *L*_NW_). As probing the longitudinal conductance, A-B oscillation amplitude of conductance may be reduced due to the diffusive transport in longitudinal direction^41^.

While, in 2014, Seung Sae Hong *et al*. from Prof. Yi Cui’s group have studied the effect of NW length on the A-B oscillations. Generally, *L*_NW_ is comparable or longer than phase coherence length (*L*_φ_). Especially at high temperature, *L*_NM_ is expected to be much longer than *L*_φ_. Therefore, if A-B oscillations are of random phase nature, oscillations of different segments (*L*_NM_ ~ *L*_φ_(T)) would be averaged out by additional factor (*L*_NM_/*L*_φ_(*T*))^−1/2^ ^42^. Then the amplitude of A-B oscillations in our experiments should be around 7.85 Ω * (4.62 μm/583 nm)^−1/2^ ≈ 2.79 Ω near 454.1 Ω because of the suppression, corresponding to 0.3491 *e*^2^/*h*, which is still much larger than our upper bond (~0.04 *e*^2^/*h*). Thus, to our detecting limit of 10^−3^ *e*^2^/*h*, there is no A-B oscillations.

The fast Fourier transform after background subtraction is also shown in Fig. 3f, which only has one pronounced peak at 0.337 T^−1^. This number is not close to the A-B oscillation frequency of 6.993 T^−1^ (~1/0.143 T) estimated from the cross-section area, which again confirms the absence of A-B oscillations. After carefully analyzing our data, we believe this oscillation could originate from the systematical errors of our system, probably because of the digital noises in our measurement system or from the universal conductance fluctuations^43^. If we changed the scanning speed or data acquisition speed, the background oscillation frequencies scale with it.

The absence of the A-B oscillations may be attributed to no conducting channels at the sidewalls, because of the weak interlayer coupling, as shown in Fig. 1c. Thus, there is no path for the Dirac electrons to travel around the perimeter of the NW. Therefore, we have provided another piece of evidence that ZrTe_5_ NW is a quasi-2D Dirac semimetal with very weak interlayer coupling, which is in a good agreement with W. Wang *et al*.’s conclusion^10^.

## Discussion

In summary, we have fabricated the ZrTe_5_ NWs devices with four-terminal geometry and measured the magnetoresistance properties under varied temperatures and angles. The angle-dependent SdH oscillations have unambiguously shown nontrivial conducting states with high carrier mobility (~85000 cm^2^V^−1^s^−1^), and they contribute up to 8.68% of the total conductance. Since the metallic properties under very low temperatures of our NWs and the non-zero Berry phase we obtained, we believe our ZrTe_5_ NWs belong to the Dirac semimetal. In addition, the negative magnetoresistance properties observed by Qiang Li *et al*.^5^ confirm again that ZrTe_5_ should be a Dirac semimetal. The absence of A-B oscillations suggests that there’s no path for the electrons to travel around the perimeter of our NWs. This together with the SdH oscillations suggest that there is only weak interlayer coupling between adjacent layers of the ZrTe_5_ NWs.

## Methods

The ZrTe_5_ crystal was grown by chemical vapor transportation (CVT) method. ZrTe5 was firstly exfoliated on scotch tape and then transferred onto 300 nm/300 μm SiO_2_/p-Si substrate. Conventional photolithography was used to pattern the ZrTe_5_ NWs into a micron-scale four-terminal device followed by a subsequent dry etching (5–15 s Ar ion etching). Four paralleled electrodes (50 nm Au) were defined by e-beam evaporation and the lift-off process. To study the 2D nontrivial conducting states of our ZrTe_5_ NWs, magneto transport measurements were conducted. A schematic diagram of the device structure is shown in Fig. 3a. The current is along the a-axis as shown by the yellow arrows. In order to study the angle-dependent and the temperature-dependent magneto transport properties, we have fabricated two devices with same geometry called S1 and S2, respectively.
